# Supplementary material for: WordCommentsAnalyzer: A windows software tool for qualitative research
Source: F1000Res. 2018 Sep 4;7:536. Originally published 2018 May 3. [Version 2] doi: 10.12688/f1000research.14819.2 (PMC6137411; doi:10.12688/f1000research.14819.2)
Supplement: Supplementary file 3 [file f1000research-7-17614-s0002.tgz › b2a79496-4ada-47c4-9682-a99ce867836d.zip!/Eight Iranian Health Journals/Journal of Research in Rehabilitation Sciences _41.docx]

© Journal of Research in Rehabilitation Sciences . This work is licensed under CC BY-NC 4.0 (https://creativecommons.org/licenses/by-nc/4.0/).

Volume & Issue: دوره 7، شماره 5: 1390 (ویژه نامه)

Title: شاخص‌های اثر سطح تماس پا در حالت استاتیک و دینامیک

http://jrrs.mui.ac.ir/index.php/jrrs/article/view/264

مقدمه: در میان روش‌های ارزیابی کف پا، پرینت کف پا ساده‌ترین روش می‌باشد. در این روش تاکنون اغلب تست‌ها به صورت تحمل وزن روی یک پا در حالت استاتیک انجام شده‌اند. بنابراین هدف از این مطالعه، دستیابی به شاخص‌های اثر سطح تماس در دو حالت تحمل وزن روی دو پا در حالت استاتیک و دینامیک بود. علاوه بر این تعیین رابطه بین شاخص‌های استاتیک و دینامیک از دیگر اهداف این تحقیق در نظر گرفته شده بود. مواد و روش‌ها: در این مطالعه کارآزمایی بالینی، تعداد 60 نفر از دانشجویان دانشگاه علوم پزشکی اصفهان در دو گروه نرمال و مبتلا به صافی کف پا شرکت داشتند. پرینت پای افراد به صورت استاتیک و دینامیک گرفته شد و پارامترهای شاخص قوس، شاخص پرینت پا و زاویه پرینت پا با استفاده از نرم‌افزار 2010 Solid work، مورد مطالعه قرار گرفت. تفاوت بین دو گروه توسط Two sample t-test و رابطه بین شاخص‌ها در حالت استاتیک و دینامیک با استفاده از آزمون Pearson correlation به دست آمد. یافته‌ها: شاخص‌های اثر سطح تماس دو گروه با یکدیگر تفاوت زیادی داشتند (05/0 > P). به علاوه بین شاخص‌ها در حالت استاتیک و دینامیک رابطه وجود داشت (808/0 < r). نتیجه گیری: شاخص‌های اثر سطح تماس را می‌توان در افراد مبتلا به صافی کف پا به منظور ارزیابی به کار برد. رابطه مشخصی بین پارامترها در حالت استاتیک و دینامیک وجود دارد. به همین علت پیشنهاد می‌شود ارزیابی پا طی راه رفتن با در نظر گرفتن پارامترهای استاتیک صورت گیرد.

Title: تعیین مهارت‌های آگاهی واج‌شناختی در کودکان مبتلا به سندرم داون دارای سن عقلی 12-7 سال آموزش‌پذیر شهر اصفهان

http://jrrs.mui.ac.ir/index.php/jrrs/article/view/297

مقدمه: کسب مهارت‌های آگاهی واج‌شناختی در رشد گفتار و زبان و تحصیل کودکان تأثیر به سزایی دارد. این مهارت‌ها باعث می‌گردد که کودکان قادر به بیان کامل‌تر مفاهیم ذهنی خویش شوند. مطالعات قبلی نشان دادند که کودکان مبتلا به سندرم داون مشکلاتی در فراگیری مهارت‌های آگاهی واج‌شناختی دارند. هدف این پژوهش، تعیین سطح مهارت‌های آگاهی واج‌شناختی کودکان مبتلا به سندرم داون بود. مواد و روش‌ها: در این تحقیق که به روش توصیفی- تحلیلی انجام شد، کلیه کودکان مبتلا به سندرم داون شهر اصفهان، با استفاده از آزمون آگاهی واج‌شناختی مورد بررسی قرار گرفتند. به منظور اجرای آزمون، آزمودنی‌ها به صورت انفرادی توسط زیرآزمون‌ها مورد ارزیابی قرار گرفتند و نتایج در چک لیست مورد نظر ثبت شد. تجزیه و تحلیل نتایج با استفاده از آزمون t-test و نرم‌افزار 16SPSS صورت گرفت. یافته‌ها: در این پژوهش هر چند دختران بهتر از پسران عمل کردند، ولی تفاوت معنی‌داری بین مهارت‌های آگاهی واج‌شناختی در دو جنس دختر و پسر مشاهده نشد. بالاترین میانگین مهارت‌های آگاهی واج‌شناختی در 12 حیطه مورد نظر، در هر دو جنس مربوط به مهارت ترکیب واج و کمترین میانگین به دست آمده، مربوط به مهارت آگاهی از هجای وسط بود. ترتیب آسان به مشکل مهارت‌های آگاهی واج‌شناختی در دو جنس به صورت؛ شناسایی واج اول، شناسایی واج آخر، آگاهی از هجای آخر، قافیه، تجانس، حذف واج اول و حذف واج میانی بود. نتیجه گیری: بر اساس نتایج پژوهش حاضر، مهارت‌های آگاهی واج‌شناختی در دختران بهتر از پسران بود (هر چند تفاوت‌ها معنی‌دار نبود). به علاوه نتایج حاصله نشان داد که همان گونه که در کودکان عادی دیده می‌شود، در گروه مورد مطالعه نیز مهارت در تمام موارد آگاهی واج‌شناختی یکسان نبوده و به یک اندازه رشد نکرده است. به عبارت دیگر برخی از موارد نسبت به بقیه مشکل‌تر بودند و زمان بیشتری برای رشد نیاز داشتند.

Title: تأثیر الگوی اصلاح شده حرکت‌درمانی با محدودیت اجباری بر کارکرد دستی کودکان دچار فلج مغزی نیمه بدن بر مبنای سطوح International classification of functioning (ICF)

http://jrrs.mui.ac.ir/index.php/jrrs/article/view/355

مقدمه: از ديدگاه طبقه‌بندي بين‌المللي کارکرد، ناتواني و سلامت (International classification of functioning, disability and health یا ICF)، فلج مغزي با نقص‌هاي ساختار و کارکرد بدني، محدوديت قابل توجه در فعاليت‌ها و مشارکت در نقش‌هاي اجتماعي همراه است. هدف این پژوهش بررسی تأثیر روش اصلاح شده حرکت‌درمانی با محدودیت اجباری بر اساس سطوح ICF بوده است. مواد و روش‌ها: این پژوهش با طرح کارآزمایی کنترل شده تصادفی (یک‌سو کور و طرح دو عاملی)، بر روی 28 شرکت کننده که بر اساس ملاک‌های ورود و کنارگذاری در دو گروه حرکت‌درمانی با محدودیت اجباری و گروه شاهد قرار گرفتند، انجام شد. مداخله در گروه حرکت‌درمانی با محدودیت اجباری به صورت 6 ساعت در روز، در طی 10 روز انجام شد. گروه شاهد از درمان رایج کاردرمانی استفاده می‌کردند. به منظور مقایسه بین دو گروه از آزمون Independent-samples t test برای اختلاف نمرات و برای بررسی تأثیر هر روش از آزمون Paired-samples t test استفاده شد. یافته‌ها: بر اساس سطوح ICF در سطح ساختار و کارکرد بدنی در متغیرهای تون عضلانی شانه، دامنه حرکتی غیر فعالانه مچ دست و ساعد بین دو گروه اختلاف معنی‌داری دیده شد (05/0 > P). همچنین در سطوح فعالیت و مشارکت نیز تفاوت‌هایی در متغیرهای چیره‌دستی، هماهنگی دوطرفه، هماهنگی اندام فوقانی و ادراک والدین (فراوانی و کیفیت) دیده شد. نتیجه گیری: از يافته‌هاي پ‍ژوهش حاضر مي‌توان نتيجه‌گيري نمود كه حرکت‌درمانی با محدودیت اجباری نسبت به سطح كاركرد و ساختار بدني بر سطوح فعاليت و مشاركت تأثير مطلوب‌تري داشته است. اين موضوع ممكن است به عواملي نظير تكليف مدار بودن روش، طول دوره و انگيزش مرتبط باشد. اين مسأله حاكي از آن است كه حرکت‌درمانی با محدودیت اجباری در مقايسه با برخي روش‌ها ( روش‌هاي فرايند مدار) تأثير بهنگام‌‌تري بر عملكرد روزمره و كيفيت زندگي كودكان به جاي می‌گذارد.

Title: بررسي اثرات تمرينات ایزومتریک در كاهش دردهاي کمر و لگن در خانم‌هاي باردار شهر اصفهان

http://jrrs.mui.ac.ir/index.php/jrrs/article/view/365

مقدمه: تغييرات فيزيولوژيك مرتبط با حاملگي باعث به هم خوردن وضعیت بدن و ايجاد كمردرد مي‌شود كه هنوز علت خاص اين كمردردها مشخص نيست. در حال حاضر به دليل تناقض‌هاي بسيار، اثرات تمريني- درماني در كاهش كمردردهاي دوران بارداري به طور كامل شناخته نشده است. هدف از اجراي اين مطالعه بررسي تأثير تمرينات ايزومتريك در كاهش دردهاي لگني و كمردرد در خانم‌هاي باردار شهر اصفهان بود. مواد و روش‌ها: نوع مطالعه کارآزمایی بالینی تصادفی (Randomized clinical trials) بود. تعداد 58 خانم باردار در ماه‌هاي بارداري بين 3 تا 7 ماه، در رده سني20 تا 35 سال مبتلا به دردهاي لگني و كمردرد كه در ماه آبان و آذر 1387 به كلينيك زنان و زايمان مراجعه كرده بودند، وارد مطالعه شدند. افراد به صورت تصادفي در دو گروه مساوي قرار گرفتند. به گروه آزمون (29 نفر) تمرینات ایزومتریک آموزش داده شد و گروه شاهد جهت هیچ مداخله‌ای قرار نداشتند. همه افراد پرسش‌نامه مربوط به كمردرد و توانايي انجام فعاليت را پر كردند. يك ماه بعد همه افراد پرسش‌نامه دوم حاوي همان اطلاعات را پر كردند. اطلاعات به دست آمده در نرم‌افزار 14SPSS تجزیه و تحلیل شد و سطح معني‌داري 05/0 > P در نظر گرفته شد. یافته‌ها: در تمام فعاليت‌هاي مورد نظر اختلاف معني‌داري بين گروه تمرين‌درماني و شاهد وجود داشت (05/0 > P). همچنين كاهش شدت درد به صورت قابل توجهي قبل و بعد از تمرين‌درماني مشاهده شد (05/0 > P). در صورتي كه در گروه شاهد اختلاف معني‌داري ديده نشد (05/0 < P). نتیجه گیری: تمرینات ایزومتریک مي‌تواند به عنوان روشي مؤثر در كاهش دردهاي كمر و لگن در دوران بارداري استفاده شود.

Title: تعیین فراوانی انواع خطاهای تولید جبرانی در کودکان 7-3 ساله مبتلا به شکاف کام

http://jrrs.mui.ac.ir/index.php/jrrs/article/view/237

مقدمه: شکاف کام و لب یکی از اختلالات ساختمانی است که بر وضوح گفتار تأثیر می‌گذارد و عملکرد ارتباطی را مختل می‌سازد. در کودکان مبتلا به شکاف کام اغلب بی‌کفایتی دریچه کامی- حلقی و به دنبال آن خطاهای تولید جبرانی انتظار می‌رود. در این پژوهش سعی شد که در زمینه درصد فراوانی انواع خطاهای تولید جبرانی در سنین پیش‌دبستانی اطلاعاتی فراهم شود تا شاید زمینه‌ای برای مطالعات بیشتر در این حیطه به وجود آید. مواد و روش‌ها: این پژوهش یک مطالعه توصیفی- مقطعی بود. با توجه به کل نمونه‌های در دسترس، تعداد 31 کودک دچار شکاف کام 7-3 ساله از کلینیک‌های گفتاردرمانی شهر اصفهان انتخاب گردید. سپس با استفاده از تست تولیدی رایج در پرونده‌های تولید و تشدید، از آزمون تکرار کلمه برای مشخص کردن نوع خطای تولید جبرانی استفاده شد. صدای کودک در حین ارزیابی با ضبط صوت ضبط گردید. سپس خطاها با شنیدن مجدد صدای کودک و اتفاق نظر کلیه آزمون‌گران ثبت شد. اطلاعات به دست آمده توسط نرم‌افزار 13SPSS مورد تجزیه و تحلیل قرار گرفت. یافته‌ها: درصد فراوانی خطای تولید جبرانی انسدادی چاکنایی در جمعیت مورد مطالعه برابر 1/58 درصد، خطای انسدادی حلقی برابر 4/48 درصد، خطای سایشی حلقی برابر 3/61 درصد، خطای مرکب حلقی برابر با 7/38 درصد، خطای سایشی خیشومی خلفی برابر با 6/22 درصد و خطای انسداد خلفی میانی کام برابر 2/74 درصد به دست آمد. نتیجه گیری: در این تحقیق درصد فراوانی انواع خطاهای تولید جبرانی به دست آمد که با تعدادی از نتایج پژوهش‌های خارجی متفاوت بود. این مسأله شاید به تفاوت در تعداد نمونه‌ها، گروه‌های سنی و طبقه‌بندی این خطاها در تحقیقات مختلف مربوط می‌شود.

Title: بررسی و مقایسه حوادث دوران رشد در کودکان مبتلا به اختلال اتیسم و کودکان طبیعی

http://jrrs.mui.ac.ir/index.php/jrrs/article/view/435

مقدمه: اتيسم ناتواني رشدي پيچيده‌ای است كه به طور شاخص در طول سه سال اول زندگي در نتيجه يك اختلال نورولوژيك تأثيرگذار بر عملكرد نمايان مي‌شود. مطالعه حاضر با هدف، بررسی فراوانی تعدادی از حوادث تأثیرگذار در دوران رشد در کودکان مبتلا به اختلال اتیسم و سپس مقایسه این حوادث در کودکان طبیعی انجام شد. مواد و روش‌ها: این پژوهش توصیفی- تحلیلی روی 50 کودک مبتلا به اختلال اتیسم و 50 کودک طبیعی 11-4 ساله شهر اصفهان انجام شد. ابتدا حوادث مهم و تأثیرگذار قبل، حین و بعد از تولد کودکان مبتلا به اتیسم با بررسی متون جمع‌آوری گردید و سپس در قالب پرسش‌نامه واحدی توسط مادر کودک تکمیل شد. نتایج به دست آمده توسط نرم‌افزار 14SPSS و سطح معنی‌داری 05/0 > P به وسیله آزمون‌های 2χ، Mann-Whitney test و Fisher's exact test مورد تجزیه و تحلیل آماری قرار گرفت. یافته‌ها: افزایش سن پدر و مادر، بالا بودن استرس مادر در دوران بارداري، وجود علایم آلرژيك در دوره بارداری، ازدواج فامیلی در والدین کودک، وجود سابقه اختلالات ارتباطی و گفتار و زبان در خانواده، زایمان مادر به صورت سزارین، تب بالا، ضربه به سر و تشنج با وقوع اختلال اتیسم رابطه معنی‌داری داشتند (05/0 > P). نتیجه گیری: اگر چه به کمک این مطالعه قادر نخواهیم بود که علل قاطعی برای اختلال اتیسم ارایه دهیم، ولی به این وسیله عوامل خطرساز احتمالی معرفی خواهند شد. علاوه بر این یافته‌های این مطالعه بیانگر این است که عوامل قبل، حین و بعد از تولد که به نوعی به انحراف در رشد جنین مربوط می‌شوند، در ایجاد اختلال اتیسم نقش مهمی دارند.

Title: تأثیر استفاده از کفی‌های طبی در میزان مصرف انرژی افراد دچار صافی کف پا طی راه رفتن

http://jrrs.mui.ac.ir/index.php/jrrs/article/view/254

مقدمه: کاهش ارتفاع قوس طولی داخلی پا تحت عنوان صافی کف پا خوانده می‌شود. تحقیقات بسیاری پیرامون اثبات تأثیر صافی کف پا بر ویژگی‌های راه رفتن افراد صورت گرفته است، اما در زمینه تأثیر کف پای صاف بر مصرف انرژی این افراد شواهد اندکی وجود دارد. هدف از مطالعه حاضر، بررسی تأثیر صافی کف پا و درمان ارتزی آن بر تغییرات مصرف انرژی این بیماران بود. مواد و روش‌ها: در این مطالعه دو گروه 25 نفری از افراد سالم و دارای کف پای صاف شرکت کردند. مطالعه از نوع کارآزمایی بالینی بود. نمونه‌های مورد نظر از بین دانشجویان و کارمندان خانم دانشگاه علوم پزشکی اصفهان با میانگین سنی 21 سال انتخاب شدند. ارزیابی هر دو پای افراد هر دو گروه از طریق فوت‌پرینت و به کمک نرم‌افزار Solid work انجام شد. برای اندازه‌گیری میزان مصرف انرژی از متغیر شاخص نرخ فیزیولوژیکی (Physiological cost index یا PCI) استفاده گردید. بررسی این متغیر با کمک سیستم اندازه‌گیری ضربان قلب در حین حرکت، ساخت شرکت پولار صورت گرفت. تفاوت بین PCI هر دو گروه توسطTwo sample t-test  و تأثیر استفاده از ارتز توسط Paired t-test مورد بررسی قرار گرفت. میزان متوسط PCI در افراد سالم 368/0 و در افراد با صافی کف پا 357/0 ارزیابی گردید (0393/0 = P). یافته‌ها: مصرف انرژی افراد دچار صافی کف پا نسبت به افراد سالم تفاوت محسوسی داشت. استفاده از کفی طبی عملکرد افراد دچار صافی کف پا را حین راه رفتن ارتقاء داد (05/0 > P). مقدار مصرف انرژی بر اساس پارامترهای PCI و THB (Total heart rate beat) تفاوت معنی‌داری را حین استفاده از کفی و بدون کفی نشان داد (05/0 > P). نتیجه گیری: راستای نا‌مطلوب مفاصل اندام تحتانی در بیماران دچار صافی کف پا موجب افزایش PCI در افراد این گروه نسبت به افراد سالم می‌شود و استفاده از کفی‌های طبی موجب بهبود راستای پا و در نتیجه کاهش مصرف انرژی در این افراد می‌گردد.

Title: تأثیر بازی‌های دبستانی منتخب بر رشد ادراکی- حرکتی و رشد اجتماعی دختران 9-8 سال

http://jrrs.mui.ac.ir/index.php/jrrs/article/view/251

مقدمه: هدف از انجام این تحقیق، بررسی تأثیر یک دوره بازی‌های منتخب دبستانی بر رشد ادراکی- حرکتی و رشد اجتماعی کودکان بوده است. همچنین تأثیر این بازی‌ها بر اجزای رشد ادراکی- حرکتی همچون سرعت، تعادل، هماهنگی دو جانبه، قدرت، هماهنگی فوقانی، سرعت پاسخ، کنترل دیداری حرکتی و چالاکی اندام فوقانی در دختران 9-8 ساله مورد بررسی قرار گرفت. مواد و روش‌ها: در اين كارآزمايي باليني تصادفي، تعداد 48 نفر از دانش‌آموزان کلاس دوم و سوم ابتدایی به صورت تصادفی از بین نمونه‌های همگن شده به وسیله پرسش‌نامه فردی انتخاب گردیدند و پس از انجام پیش‌آزمون مهارت‌های ادراکی- حرکتی Bruininks–Oseretsky و آزمون رشد اجتماعی Vineland به دو گروه بازی‌های دبستانی و فعالیت‌های معمول تقسیم شدند. در مرحله بعد بازی‌های دبستانی به مدت 8 هفته و 3 جلسه در هر هفته اجرا شد. در نهایت از هر دو گروه پس‌آزمون به عمل آمد. سپس اطلاعات به دست آمده از پرسش‌نامه مشخصات فردی و آزمون‌های مربوط ثبت و به صورت توصیفی طبقه‌بندی و تنظیم گردید. آناليز داده‌ها با استفاده از نرم‌افزار 16SPSS و در سطح 05/0 > P صورت گرفت. تجزیه و تحلیل اطلاعات با استفاده از آزمون‌های پارامتریک Independent t-test و Dependent t-test انجام شد. یافته‌ها: بازی‌های منتخب دبستانی نسبت به فعالیت‌های معمول از نظر آماری تأثیر معنی‌داری بر سرعت، هماهنگی فوقانی، چالاکی اندام فوقانی و رشد ادراکی- حرکتی آزمودنی‌ها داشت (001/0 > P). ولی تأثیر آن بر تعادل ایستا و پویا، هماهنگی دو جانبه، قدرت، سرعت پاسخ، کنترل دیداری- حرکتی و رشد اجتماعی آزمودنی‌ها معنی‌دار نبود (05/0 < P). نتیجه گیری: بازی‌های دبستانی می‌تواند برنامه مناسبی برای رشد مهارت‌های ادراکی- حرکتی کودک باشد. این در حالی است که ارتقای مهارت‌های اجتماعی کودکان نیازمند فعالیت‌های گروهی برنامه‌ریزی شده و ساختارمند می‌باشد.

Title: بررسی همخوانی آزمون شناختی مونترآل با آزمون معاینه مختصر وضعیت روانی در سالمندان سالم

http://jrrs.mui.ac.ir/index.php/jrrs/article/view/330

مقدمه: آسیب شناختی خفیف (Mild cognitive impairment یا MCI) یک وضعیت بینابینی میان وضعیت شناختی طبیعی سالمندی و دمانس می‌باشد و سندرم بالینی است که در گروه آسیب‌های شناختی قرار می‌گیرد. سالمندان مبتلا به MCI خطر پیشرفت بیماری را تا دمانس و به خصوص بیماری آلزایمر دارند. با توجه به روند پیر شدن جامعه ضرورت شناسایی ابزارهایی که بتواند با روشی آسان موارد مشکوک به این اختلال را شناسایی کنند، به شدت احساس می‌شود. این مطالعه با هدف بررسی همخوانی بین دو آزمون MMSE (Mini-Mental state examination) و MoCA (Montreal cognitive assessment) در تعیین اختلال شناختی در سالمندان سالم که علایم بالینی اختلال شناختی خفیف را نشان نمی‌دهند، طراحی و انجام شده است. مواد و روش‌ها: در این مطالعه مقطعی 47 سالمند (25 زن و 22 مرد) با سن 60 سال به بالا در سطح شهر شیراز از طريق نمونه‌گيري در دسترس، مورد بررسی قرار گرفتند. از افراد شرکت کننده آزمون‌های MMSE و MoCA به صورت آزمون- پس‌آزمون و با فاصله 2 ماه گرفته شد و روش‌های آماری به کار گرفته شده جهت مقایسه همخوانی این دو آزمون شامل Regression analysis بود. یافته‌ها: میانگین سنی افراد سالمند 05/22 ± 43/69 بود. ضریب همبستگی Pearson (0001/0 > P، 69/0 = r) رابطه معنی‌دار مثبتی بین آزمون MoCA درtest  و retest نشان داد. عدم وجود همخوانی بین دو آزمون MMSE و MoCA توسط تحلیل رگرسیون ساده (602/0 = r و 362/0 = 2R) نشان داده شد. نتیجه گیری: نتایج نشان داد که بین دو آزمون همخوانی قابل قبولی وجود ندارد. به نظر می‌رسد به دلیل بالا بودن ثبات داخلی آزمون ارزیابی شناختی مونترآل و سالم بودن جمعیت مورد مطالعه می‌باشد. عدم همخوانی بین نمرات این دو آزمون به معنی عدم توانایی آزمون معاینه مختصر وضعیت روانی در تشخیص اختلال شناختی خفیف شناختی می‌باشد.

Title: بررسی اندازه خزانه واژگان بیانی کودکان طبیعی 36-18 ماهه فارسی زبان

http://jrrs.mui.ac.ir/index.php/jrrs/article/view/190

مقدمه: فراگیری واژگان یک مؤلفه پیچیده و اصلی از رشد زبانی کودک در مراحل اولیه زبان آموزی می‌باشد. هدف از پژوهش حاضر، تعیین اندازه خزانه واژگان بیانی کودکان 36-18 ماهه طبیعی بود تا از طریق نتایج آن اطلاعاتی در مورد معیارهای اولیه رشد خزانه واژگان بیانی به دست آید. مواد و روش‌ها: این مطالعه توصیفی- مقطعی بود. اندازه خزانه واژگان بیانی 42 کودک 36-18 ماهه طبیعی در سه گروه سنی با فواصل 6 ماهه با استفاده از فرم II از فهرست CDI مورد ارزیابی قرار گرفت. فرم‌ها توسط مادران تکمیل شد و تعداد واژگان بیانی هر کودک به طور جداگانه محاسبه گردید و با استفاده از آزمون‌های آنالیز واریانس تحلیل شد. یافته ها: میانگین اندازه خزانه واژگان بیانی در سه گروه سنی از نظر آماری متفاوت بود (001/0 > P). در سه گروه سنی میانگین اندازه خزانه واژگان بیانی، طبقه اسامی رایج بیشترین (001/0 = P) و واژگان دستوری کمترین سهم (02/0 = P) را از اندازه خزانه واژگان بیانی داشتند. نتیجه گیری: یافته‌ها بیان‌گر وجود تنوع زیاد در تعداد واژگان طبقات واژگانی مختلف در این محدوده سنی بود. اسامی رایج به علت کاربرد زیاد در محیط، بیشترین سهم و واژگان دستوری به علت بسامد و ضرورت کم در استفاده، کمترین سهم از واژگان را در بر گرفته بود.

Title: ترجمه و معادل‌سازي پرسش‌نامه «مقياس سنجش تأثيرات سكته مغزي» و بررسي روايي نسخه فارسي در افراد مبتلا به سكته مغزی

http://jrrs.mui.ac.ir/index.php/jrrs/article/view/347

مقدمه: هدف از پژوهش حاضر، فراهم‌‌سازي ابزاري با روايي مطلوب به زبان فارسي، جهت سنجش تأثيرات سكته مغزي در افراد مبتلا به سكته مغزي (Stroke) از طريق ترجمه و معادل‌سازي پرسش‌نامه SIS (Stroke impact scale) به زبان فارسي و بررسي روايي (Validity) نسخه فارسي معادل‌سازي شده در این افراد بود. مواد و روش‌ها: تحقيق حاضر يك مطالعه غير تجربي از نوع متدولوژيك بود. پرسش‌نامه SIS طي سه مرحله طبق روش IQOLA (International quality of life assessment project) به زبان فارسي ترجمه و معادل‌سازي شد. در اين تحقيق، روايي صوري (Face validity)، روايي همگرا (Convergent validity) و روايي همگرايي گزینه‌ها (Item convergence) بررسي گرديد. در پژوهش حاضر جهت بررسي روايي همگرا، 20 نفر از افراد مورد مطالعه توسط نسخه فارسي پرسش‌نامه SIS ارزیابی گردیدند و دوباره همان افراد توسط ساير مقياس‌هاي خارجي معتبر FMA (Fugal meyer motor assessment)، SF-36 (Social functioning)، FAT (Farsi aphasia test)، MMSE ( Mini mental state examination)، HADS (Hospital anxiety depression scale) و BI (Barthel ADL index) مورد ارزيابي قرار گرفتند. همچنین جهت بررسی روايي همگرايي گزینه‌ها، این پرسش‌نامه در اختیار 60 نفر از افراد مورد مطالعه قرار گرفت و ضریب همبستگی نمره هر گزینه با نمره کل همان حوزه تحت تجزیه و تحلیل آماری قرار گرفت. كلیه افراد مورد مطالعه (80 = n) طبق تشخيص پزشك، مبتلا به سكته مغزي بودند و به دو بيمارستان كاشاني و الزهرا (س) در شهر اصفهان از سال 1384 تا 1386، جهت دريافت خدمات پزشكي و توان‌بخشي مراجعه كرده بودند. روش نمونه‌گيري به صورت ساده و غير احتمالي بود. یافته‌ها: سؤالات از نظر بیماران قابل قبول و قابل فهم گزارش شد. ضرايب همبستگي بين هر يك از حوزه‌های نسخه فارسي معادل‌سازي شده پرسش‌نامه SIS با مقياس خارجي استاندارد به كار گرفته شد، بين 67 تا 96 درصد را نشان مي‌داد. لذا مقیاس‌های خارجی به کار گرفته شده است و آزمون SIS هر دو به بررسی یک مفهوم و بررسی ساختار مورد نظر می‌پردازند. ضرایب همبستگي بالا بين نمره هر گزینه با نمره كل هر حوزه مربوطه، نمايان‌گر این مهم است که هر گزینه در مجموع گزینه‌های حوزه مربوطه مناسب بود و همگی به بررسی یک ساختار مشترک می‌پردازند. نتیجه گیری: پرسش‌نامه SIS از روايي خوبی برخوردار بود و مي‌توان آن را به عنوان ابزاری روا، به منظور سنجش تأثیرات بیماری در افراد مبتلا به سکته مغزی به کار برد.

Title: تعيين تأثير طب سوزني ليزري بر درمان افراد مبتلا به لكنت و مقايسه آن با گفتار‌درماني

http://jrrs.mui.ac.ir/index.php/jrrs/article/view/286

مقدمه: بررسي استراتژي و روش‌هاي درماني جايگزين در درمان لكنت با توجه به ويژگي‌هاي اين اختلال و طولاني بودن زمان درماني و احتمال بازگشت آن، از اهميت زيادي برخوردار است. طب سوزني به عنوان يك روش درماني در درمان لکنت، معرفي شده است. هدف از انجام این پژوهش، تعيين نتايج مداخله طب سوزني ليزري و مقايسه آن با گفتار‌درماني بر درمان افراد مبتلا به لكنت بود. مواد و روش‌ها: این مطالعه تحلیلی و به شیوه مورد- شاهدی بر روی 20 فرد دارای لکنت و 20 فرد فاقد لکنت انجام شدگرفت. در اين پژوهش همراه با گفتاردرماني، طب سوزني ليزری بر روي 10 نفر از افراد مبتلا به لكنت كه از كودكي دچار لكنت بودند، استفاده شد. سپس نتايج با يافته‌هاي حاصل از درمان 10 نفر افراد مبتلا به لکنت كه فقط تحت گفتاردرماني و لیزر‌درمانی دارونما قرار داشتند، مقايسه شد. تمام نمونه‌ها 12 هفته پس از پايان مداخله پی‌گیری شدند. داده‌ها با استفاده از آزمون‌های تحلیل واریانس یک طرفه مکرر و آزمون t مستقل و نرم‌افزار 18SPSS مورد تجزیه و تحلیل قرار گرفتند. یافته‌ها: همراه بودن گفتاردرماني با طب سوزني ليزري باعث افزايش تأثير‌گذاري درمان شده است و ثبات و باقي ماندن درمان نيز افزايش مي‌يابد و احتمال برگشت لكنت پس از درمان نيز كاهش مي‌يابد. سرعت گفتار و درصد کلمات لكنت شده قبل و پس از درمان در هر دو گروه الف و ب كاهش نشان داد، اما پي‌گيري درمان پس از 12 هفته نشان داد كه در گروه طب سوزني ليزري، نتايج ثبات درماني بيشتري داشت و تفاوت ميان دو گروه معني‌دار است. نتیجه گیری: نتایج نشان داد که استفاده از طب سوزنی لیزری یا لیزر درمانی همراه با گفتار درمانی تأثیرات زیادی بر درمان لکنت داشته و از عود یا بازگشت لکنت پس از پایان درمان که یک مسأله بسیار شایع است، نیز تا حد زیادی جلوگیری می‌کند.

Title: مروری بر تأثیر یک دوره تمرینات توان‌بخشی بر عوامل فیزیولوژیک در افراد مبتلا به بیماری قلبی عروقی

http://jrrs.mui.ac.ir/index.php/jrrs/article/view/292

مقدمه: بیماری‌های قلبی عروقی، یکی از بزرگ‌ترین مشکلات جوامع امروزی است که درصد زیادی از بیماری‌زایی و مرگ و میر را به خود اختصاص داده است. از آن جا که فعالیت فیزیکی یا به عبارتی ورزش، در درمان و پیش‌گیری از بیماری‌های مختلف به خصوص بیماری‌های قلب و عروق نقش به سزایی ایفا می‌کند، امروزه علم جدید جهت برآوردن این امر در بطن برنامه‌های درمانی و پزشکی کوشش فراوانی نموده است. از آن جمله تأسیس مراکز توان‌بخشی قلبی می‌باشد که تمرینات ورزشی در قالب برنامه توان‌بخشی زیر نظر پزشک، روی بیماران قلبی به اجرا در می‌آیند. از این رو، محقق بر آن است تا با مرور بر شواهد علمی، اثرات مفید برنامه توان‌بخشی قلبی و انجام تمرینات ورزشی متعدد را، بر بازگشت بیماران قلبی عروقی به زندگی فعال و رضایت‌مندانه، ارایه نماید. مواد و روش‌ها: مراکز توان‌بخشی قلبی با ارایه خدمات جامع و فراگیر از جمله ارزیابی پزشکی، تجویز تمرینات ورزشی، اصلاح ریسک فاکتورهای قلبی، آموزش و مشاوره به منظور پیش‌گیری اولیه و ثانویه این بیماری طراحی شده است و بیماران قلبی می‌توانند با گذراندن چهار مرحله از مراقبت‌های توان‌بخشی که تحت نظارت پزشک، فیزیولوژیست ورزش و پرستار انجام می‌گیرد، از اثرات مفید آن به طور مستمر بهره‌مند گردند. نتیجه گیری: توان‌بخشی قلبی به همراه تمرینات ورزشی منظم، در متوقف نمودن روند بیماری‌های قلبی و کاهش علایم وابسته به آن و بهبود سلامت روانی و افزایش ظرفیت عملکردی و برگشت به زندگی مجدد بدون وابستگی به دیگران و از همه مهم‌تر کاهش مرگ و میر نقش دارد.

Title: نگاهی بر مدل نوروفیزیولوژیکی وزوز

http://jrrs.mui.ac.ir/index.php/jrrs/article/view/345

مقدمه: وزوز یکی از علایم شایع در بیماری‌های گوش و آسیب‌های نورولوژیک است که می‌توان آن را یک علامت پزشکی دانست که با وجودی که هیچ صدایی در محیط وجود ندارد، بیمار از وجود صدا در گوش یا سر شکایت دارد. این شکایت اغلب به عنوان یک بیماری در نظر گرفته نمی‌شود، بلکه خودش بخشی از شرایط بیماری مانند کم‌شنوایی ناگهانی، ضربه صوتی، اختلال عملکرد حلزونی، بیماری منیر یا اختلال نورولوژیک است. تجزیه پدیده وزوز از دیدگاه علوم اعصاب موجب ایجاد یک نظریه جدید درباره وزوز شده است که در این مقاله با جزییات کامل به عنوان مدل نوروفیزیولوژیک وزوز توضیح داده شده است. مواد و روش‌ها: با استفاده از کلید واژه‌های "وزوز"، "مدل نوروفیزیولوژیک وزوز" و "مکانیسم‌های وزوز" مقالات منتشر شده در این مورد، در بانک‌های اطلاعاتی Science direct، Pubmed، Google scholar، Proquest و Scopus در بازه زمانی 2011-1994، مورد بررسی قرار گرفت. یافته‌ها: حاصل جستجوی انجام گرفته، 35 مقاله پژوهشی و 7 مقاله مروری بود. پس از بررسی عنوان، مقدمه و بحث در مورد مقالات پژوهشی و مطالعه کامل مقالات مروری، 18 مقاله یافت شد که به موضوع مورد نظر پرداخته بودند و با استفاده از آن‌ها این مقاله مروری نگاشته شد. نتیجه گیری: بر اساس مدل نوروفیزیولوژی وزوز مسیرهای شنوایی و غیرشنوایی در ایجاد وزوز نقش دارند. درک بلندی وزوز و میزان آزاردهندگی به فعالیت سیستم لیمبیک بستگی دارد.

Title: کار (Occupation) انسانی: اولین کوشش در تعریف تشریحی آن

http://jrrs.mui.ac.ir/index.php/jrrs/article/view/331

مقدمه: کار «Occupation» شامل تکالیف روزمره و فعالیت‌های هدفمندی است که در آن‌ها مشارکت می‌کنیم و برای هر فرد معنی و یا ارزش شخصی یا ذهنی خاصی داشته است و تحت تأثیر پیشینه فرهنگی، علایق و جنبه‌هایی از زندگی که برای فرد معنی دارند، قرار دارد. مفهوم «کار» به عنوان هسته مرکزی کاردرمانی، از اهمیت خاصی برخوردار می‌باشد. مفهوم «کار» دارای ساختاری پیچیده است که شامل مفاهیم مرتبط فراوانی می‌باشد که هر کدام نیز در جای خود پیچیده هستند. با توجه به تعاریف متعدد کار به نظر می‌رسد یک تعریف جامع و واحد از آن، جهت استفاده کاردرمانگران در تیم توان‌بخشی و برای شرح خدمات خود، لازم و ضروری است. مواد و روش‌ها: برای این پژوهش، روش کیفی پدیدارشناختی انتخاب شد؛ به این دلیل که هدف اصلی آن دستیابی به یک تعریف جامع در رابطه با کار بود. جامعه مورد بررسی شامل متخصصین کاردرمانی بود که از نمونه‌گیری در دسترس استفاده گردید. در مرحله اول، منابع و تعاریف موجود بررسی شد و تعریفی که در برگیرنده تمام جنبه‌های «کار» باشد تدوین گردید. سپس این تعریف توسط یک گروه از متخصصین کاردرمانی در قالب گروه متمرکز تکمیل‌ شد و جمع‌آوری داده‌ها به روش دلفی صورت گرفت. یافته‌ها: مفهوم کار دارای مفاهیم گسترده‌ای است که بر طبق نظر شرکت‌کنندگان مفاهیمی همچون هدفمندی و معنی‌داری از مؤلفه‌های اصلی آن می‌باشد. همچنین در تعریف کار باید انجام دادن فرد یا عملکرد کاری و بافتاری که کار در آن انجام شده است، مورد توجه قرار گیرند. نتیجه گیری: در تعریف ارایه شده در نتیجه این پژوهش، کار دارای ویژگی‌هایی همچون هدفمندی و معناداری است که از ارتباط بین ساختار رشدی فرد و قالب کاری او منتج گردیده است و عملکرد فیزیکی یا ذهنی شخص در حیطه‌هایی همچون فعالیت‌های روزمره زندگی، خواب و استراحت، آموزش، شغل، بازی و اوقات فراغت، مشارکت اجتماعی و یا ترکیبی از این‌ها می‌باشد. این تعریف در مقایسه با تعاریفی که تاکنون در مورد کار ارایه شده، کامل‌تر و عینی‌تر است و تمام مفاهیم ضروری و اصلی کار را در بردارد که کاردرمانگران می‌توانند از آن در شرح خدمات کاردرمانی و نیز در روند کار تیمی و در ارتباط با همکاران توان‌بخشی استفاده نمایند.

Title: مروری بر آسیب‌های زبانی در افراد مبتلا به مولتیپل اسکلروز

http://jrrs.mui.ac.ir/index.php/jrrs/article/view/313

مقدمه: مولتیپل اسکلروز (Multiple sclerosis یا MS) یکی از شایع‌ترین بیماری‌های سیستم عصبی در انسان است که اغلب بالغین جوان را مبتلا می‌سازد. در این بیماری علاوه بر علایم شایع نظیر ضعف اندام‌ها، سرگیجه و دوبینی در 50-40 درصد بیماران، اختلالات حرکتی گفتار به صورت دیزآرتری دیده می‌شود. به دلیل این که جایگاه ضایعه در این بیماری به طور عمده نواحی زیرقشری می‌باشد، این فرضیه وجود دارد که عملکرد زبانی دست نخورده باقی می‌ماند و بنابراین در برنامه‌های درمانی مورد ارزیابی و درمان قرار نمی‌گیرد. با توجه به اهمیت زبان در ارتباطات انسانی و کیفیت زندگی، این مطالعه با هدف بررسی وجود اختلالات زبانی در بیماران MS انجام شد. مواد و روش‌ها: این مقاله مروری بر تحقیقات منتشر شده بین سال‌های 2011-1990 در پایگاه‌های اطلاعاتی Pubmed، Ebsco، ISI، Elsevior و Iran doc و هم چنین جستجو در منابع کتابخانه‌ای بوده است. جستجوی اولیه با کلید واژه‌های "Rehabilitation cognition، Speech، Language و Multiple sclerosis" انجام شد. معیار گزینش مقالات در مرحله دوم شیوه اجرای پژوهش بود. به این ترتیب که با مطالعه خلاصه مقالات در صورتی که پژوهش‌گر در شیوه اجرای پژوهش خود به بررسی مهارت‌های گفتاری و زبانی مختلف در بیماران MS پرداخته بود یا در بررسی مهارت‌های شناختی افراد مبتلا به MS به مهارت‌های زبانی آن‌ها نیز توجه کرده بود، مقاله مورد نظر انتخاب و با مطالعه متن کامل آن، جهت تهیه این مقاله مورد استفاده قرار می‌گرفت. یافته‌ها: از مجموع 78 مقاله به دست آمده از جستجوی اولیه، 28 مقاله بر اساس معیار ذکر شده انتخاب گردید و متن کامل آن‌ها مطالعه شد. نتیجه‎گیری: پژوهش‌های منتشر شده نشان داد که در بیماران مبتلا به MS انواع آسیب‌های زبانی شامل نقایص نامیدن، روانی کلامی، اشکال در عملکردهای سطح بالای زبانی و مشکلاتی در سازمان‌دهی به سخنرانی وجود دارد که بایستی در ارزیابی و درمان آن‌ها مورد توجه قرار گیرد.

Title: اختلالات حافظه‌ کاری در بیماران مبتلا به اختلال اسکیزوفرنیا و روش‌های ارزیابی آن

http://jrrs.mui.ac.ir/index.php/jrrs/article/view/333

مقدمه: اختلال اسکیزوفرنیا یکی از اختلالات پیچیده و با علل چندگانه در میان اختلاات روان است و آسیب حافظه از ویژگی‌های بسیار مهم عصب‌شناختی در این بیماران است. نقص عملکرد حافظه کاری می‌تواند بسیاری از علایم اصلی در این بیماران را تحت تأثیر قرار دهد چرا که در ذخیره اطلاعات به صورت کوتاه مدت و دستکاری و بازیابی آن‌ها نقش عمده دارد. در این مطالعه سعی بر آن است تا ابعاد مختلف آسیب حافظه کاری و آزمون‌های مختلف در این خصوص در این بیماران مورد بررسی قرار گیرد. مواد و روش‌ها: در این مقاله، مروری از بانک‌های اطلاعاتی مانند PubMed، ProQuest، Science Direct و Scopus مرتبط استفاده شد. حدود 100 مقاله مرتبط در این رابطه یافت شد که 90 موردآن به زبان انگلیسی بود و 41 مقاله به صورت متن کامل و در سال‌های 1990 تا 2011 به چاپ رسیده بودند، البته مقالاتی که ملاک‌های ورود لازم را نداشتند، حذف شدند. از جمله مهم‌ترین ملاک‌های ورود، اصیل بودن و انواع مروری بودن مقالات، سال انتشار آن‌ها، مقایسه‌ای بودن و استفاده از آزمون‌های ارزیابی معتبر بود. در این مطالعه جهت تحقیق از کلمات کلیدی «اسکیزوفرنیا و حافظه، حافظه کاری، حافظه کوتاه مدت، ارزیابی حافظه و اسکیزوفرنیا و حافظه کاری، اسکیزوفرنیا و ارزیابی حافظه، حافظه کاری و ارزیابی» و مجموعه‌ای از این کلمات استفاده شد. نتیجه گیری: نتایج این مطالعه مروری حاکی از آن است که مشکلات حافظه کاری درتمام قسمت‌های آن در بیشتر بیماران اسکیزوفرن دیده می‌شود که می‌تواند بر ایجاد علایم مثبت و منفی در این بیماران تأثیر بگذارد. تحقیقات صورت گرفته از تکالیف و آزمون‌های متفاوتی جهت بررسی حوزه‌های مختلف حافظه کاری مانند لوپ صوتی و بخش دیداری- فضایی و اجرایی- مرکزی استفاده می‌کنندکه بستگی به اهداف مطالعه و بخش مورد بررسی در حافظه کاری متفاوت است و شدت علایم در نتیجه این آزمون‌ها بسیار تأثیر‌گذار است.

Title: مطالعه مروری؛ مناطق مرده حلزون و آزمون Threshold Equalizing Noise

http://jrrs.mui.ac.ir/index.php/jrrs/article/view/344

مقدمه: مناطق مرده قسمتی از حلزون در گوش داخلی است که تخریب شده‌اند. با دیدن ادیوگرام، پاسخ‌هایی دیده شده است که فرد در آن‌ها به تحریک آکوستیکی پاسخ داده است. این علایم یکپارچگی قسمتی از گوش که مورد آزمایش قرار گرفته است را نشان می‌دهد. آگاهی از عملکرد حلزون، در تشخیص پاسخ‌های کاذبی که از مناطق مرده حلزونی می‌آیند، کمک کننده است. در سال‌های اخیر به‍ وسیله آزمونی به نام TEN (Threshold equalizing noise) با ارایه نویز پهن باند همان طرفی و تغییر آستانه یک منطقه، به شناسایی مناطق مرده حلزونی پرداخته‌اند. با توجه به حساسیت مناطق مرده در دریافت و درک گفتار، آگاهی از وضعیت سلامت حلزون و نحوه انجام آزمون مربوط ضروری به نظر می‌رسد. مواد و روش‌ها: این مقاله مروری بر مقالات منتشر شده از سال 1993 تا 2003 بود که بیش‍ترین مطالعات انجام شده در این دوره زمانی و در زمینه مناطق مرده حلزون بوده است که در پایگاه‌‍‌های اطلاعاتی Google Scholar، Science Direct، Ebsco، PubMed، Thieme، Proquest و با استفاده از کلید واژه‌های مناطق مرده حلزونی، آزمون آستانه برابری شده با نویز، پوشش همان طرفی و موج مسافر در حلزون، تجویز سمعک بر اساس آستانه واقعی وجود داشت. نتیجه گیری: در تنظیم سمعک برای بیمارانی با افت شنوایی شدید و شیب‌دار باید به بخش‌های عملکردی و مناطق مرده محدوده فرکانسی شنوایی آن‌ها توجه داشت. هدف، ایجاد تقویت برای بخش‌های فعال و جلوگیری از تقویت برای بخش‌های مرده بود. اغلب این به معنی ایجاد تقویت برای فرکانس‌های مناطق گذار (مناطق بین مناطق مرده و سالم) به جای تقویت فرکانس‌هایی بود که بیش‌ترین کم‌شنوایی را داشتند. می‌توان به بخش‍‌‌های در حال مرگ کمک کرد ولی به مناطق مرده نمی‌توان تقویتی اعمال نمود. شناسایی مناطق مرده به تنظیم سمعک کمک می‌کند.

Title: بررسي رویکرد خانوده محور در توان‌بخشی کودکان دچار فلج مغزی

http://jrrs.mui.ac.ir/index.php/jrrs/article/view/361

مقدمه: کودکان دچار فلج مغزی مجموعه‌ای از مداخلات توان‌بخشی را به منظور تسهیل رشد و بهبود استقلال کارکردی در حرکت، مراقبت از خود، بازی و فعالیت‌های مدرسه و اوقات فراغت دریافت می‌کنند. کاردرمانگران در مواجهه با کودکان با اختلال فلج مغزی از رویکردهای گوناگونی بهره می‌گیرند، که در الگوی سنتی رایج‌ترین آن درمان عصبی رشدی است. امروزه با گسترش حوزه نفوذ فلسفه خانواده محور و ضرورت التفات به طبقه‌بندی بین‌المللی کارکرد، ناتوانی و سلامت و اصول مربوط به آن، محدودیت‌های رویکردهای سنتی رایج و پیشرفت‌هایی که نظریه سامانه‌های پویا ایجاد نموده است، نیاز به رویکردی نوین در کاردرمانی را ضروری می‌نماید. هدف مطالعه حاضر، توصیفی مختصر از فلج مغزی و نقش کاردرمانی در رابطه با آن، بررسی رویکرد خدمات خانواده محور، نظریه سامانه‌های پویا و رویکرد درمان کارکردی خانواده محور و کاربرد آن در توان‌بخشی کودکان دچار فلج مغزی بود. مواد و روش‌ها: در اين بررسي، متون شواهد پژوهشی که بر مبنای طبقه‌بندی درمان مبتنی بر شواهد در سطوح اول، دوم و سوم شواهد جای می‌گرفتند، وارد مطالعه شدند. مقالاتي كه در سال‌هاي 1966 تا 2011 به چاپ رسيده بود با استفاده از کلید واژه‌های Functional،  Cerebral palsy، Family-centered و Therapy در پایگاه‌های اطلاعاتی PubMed، Scopus، ProQuest و Scholar Google جستجو و سپس مقالات یافت شده بر مبنای ملاک‌های ورود و خروج انتخاب شدند و مورد بررسی قرار گرفتند. یافته‌ها: با توجه به کلید واژه‌های انتخاب شده، تعداد 128 مقاله یافت شد که پس از مطالعه چکیده مقالات، در مرحله بعد تعداد 22 مقاله بر مبنای ملاک‌های ورود مرتبط تلقی شد. در نهایت پس از مطالعه، 6 مقاله دقیقاً پاسخ‌گوی پرسش مطالعه بود. نتیجه گیری: مطالعات بررسي شده نشان دهنده آن است که رویکرد درمان کارکردی خانواده محور که تلفیقی از اصول نظری کاردرمانی، رویکرد خانواده محور و دیدگاه سامانه‌های پویا در رشد حرکت است، می‌تواند به عنوان رویکردی مناسب در توان‌بخشی کودکان دچار فلج مغزی استفاده شود. اگر چه جهت اثبات اثربخشی آن نیاز به پژوهش‌های بیشتری وجود دارد.

Title: اختلالات دهلیزی در کودکان

http://jrrs.mui.ac.ir/index.php/jrrs/article/view/342

مقدمه: از آن جا که کودکان قادر به بیان ویژگی مشکل خود نیستند، تشخیص و مدیریت اختلالات تعادلی در دوران کودکی، سخت‌تر از بزرگ‌سالان است. علایم اختلالات دهلیزی در کودکان به آسانی با مشکلات رفتاری یا پزشکی دیگر اشتباه گرفته می‌شود و موجب تشخیص نادرست و بررسی‌ها و درمان‌های نامناسب می‌گردد. بنابراین در این مطالعه مروری به بیان ویژگی‌های اختلالات دهلیزی در کودکان پرداخته شد. مواد و روش‌ها: با استفاده از کلید واژه‌های "اختلالات دهلیزی کودکان"، "منگی"و "سرگیجه" مقالات منتشر شده در این مورد، در بانک‌های اطلاعاتی Science Direct، PubMed، Google Scholar، ProQuest و Scopus در بازه زمانی 2011-1993، مورد بررسی قرار گرفت. یافته‌ها: حاصل جستجوی انجام گرفته، 85 مقاله پژوهشی و 8 مقاله مروری بود. پس از بررسی عنوان، مقدمه و بحث در مورد مقالات پژوهشی و مطالعه کامل مقالات مروری، 26 مقاله یافت شد که به موضوع مورد نظر پرداخته بودند و در این مطالعه مروری مورد بررسی قرار گرفتند. نتیجه گیری: اختلالات دهلیزی کودکان ممکن است شایع‌تر از آن چیزی باشد که پیش از این تصور می‌شد. حملات سرگیجه ممکن است به سختی تحمل شود و برای کودکان رنج‌آور باشد. بنابراین مدیریت این جنبه‌ها نیز از اهمیت زیادی برخوردار است و مشاوره لازم باید به والدین و کودک داده شود.
